# Normal observers show no evidence for blindsight in facial emotion perception

**DOI:** 10.1093/nc/niaa023

**Published:** 2020-12-12

**Authors:** Sivananda Rajananda, Jeanette Zhu, Megan A K Peters

**Affiliations:** Department of Bioengineering, University of California Riverside, Riverside, CA 92521, USA; Department of Psychology, University of California Los Angeles, Los Angeles, CA 90095, USA; Department of Bioengineering, University of California Riverside, Riverside, CA 92521, USA; Department of Cognitive Science, University of California Irvine, Irvine, CA 92697, USA

**Keywords:** blindsight, featural blindsight, emotion perception, fear detection, Bayesian ideal observer, metacognition, consciousness

## Abstract

Some researchers have argued that normal human observers can exhibit “blindsight-like” behavior: the ability to discriminate or identify a stimulus without being aware of it. However, we recently used a bias-free task to show that what looks like blindsight may in fact be an artifact of typical experimental paradigms’ susceptibility to response bias. While those findings challenge previous reports of blindsight in normal observers, they do not rule out the possibility that different stimuli or techniques could still reveal perception without awareness. One intriguing candidate is emotion processing, since processing of emotional stimuli (e.g. fearful/happy faces) has been reported to potentially bypass conscious visual circuits. Here we used the bias-free blindsight paradigm to investigate whether emotion processing might reveal “featural blindsight,” i.e. ability to identify a face’s emotion without introspective access to the task-relevant features that led to the discrimination decision. However, we saw no evidence for emotion processing “featural blindsight”: as before, whenever participants could identify a face’s emotion they displayed introspective access to the task-relevant features, matching predictions of a Bayesian ideal observer. These results add to the growing body of evidence that perceptual discrimination ability without introspective access may not be possible for neurologically intact observers.

## Introduction

The neurological condition of blindsight has fascinated consciousness researchers since its discovery (Weiskrantz, 198 6, 1996; Azzopardi and Cowey 1997, 1998); in this rare condition, patients with damage to primary visual cortex can directly discriminate some aspects of visual stimuli but report no conscious visual awareness of them. Thus, to experimentally use this dissociation to isolate conscious awareness of a stimulus from the potential confound of signal processing capacity (Lau, 2008; Morales *et al.* 2015; Giles *et al.* 2016; Peters *et al.* 2017b), we must find a way to induce blindsight-like behavior in neurologically intact observers. Note that, we aim to induce absolute blindsight, where consciousness awareness is abolished completely, as opposed to relative blindsight, where performance is matched while confidence differs (Lau and Passingham 2006; Balsdon and Azzopardi 2015; Peters and Lau 2015; Peters *et al.* 2017a; Knotts *et al*., 2018). Successful induction of such “performance without awareness” would pave the way for computational and neuroimaging studies seeking to identify the neural correlates of consciousness (Rees *et al.* 2002; Block, 2005; Tononi and Koch 2008; Aru *et al.* 2012).

To measure unconscious perception, researchers have generally been divided on two approaches: those relying on the *objective threshold*, and those relying on the *subjective threshold* as the relevant criterion for awareness of a stimulus (c.f. Snodgrass and Shevrin 2006). Objective threshold advocates suggest that performance at chance (*d*′ = 0) is the threshold at which the subject becomes conscious of the stimulus (i.e. the subject is conscious of the stimulus if *d*′ > 0). Subjective threshold advocates suggest that the threshold at which a stimulus becomes conscious is the same threshold at which they are able to report it (i.e. only if the subject reports that they saw the stimulus are they conscious of it, meaning *d*′ > 0 does not necessarily indicate consciousness of the stimulus).

Researchers relying on the subjective threshold definition for stimulus awareness have often attempted to induce blindsight-like behavior in normal observers, typically using visual masking or other similar techniques. Unfortunately, most of these attempts are susceptible to response bias confounds (Eriksen, 1960; Merikle *et al.* 2001; Ramsøy and Overgaard 2004; Hannula *et al.* 2005; Charles *et al.* 2013, 2014; Lloyd *et al.* 2013; Jachs *et al.* 2015; Peters *et al.* 2016, 2017a). Further, past attempts to remedy response bias concerns (Kolb and Braun 1995; Kunimoto *et al.* 2001) have met with their own conceptual or replicability challenges (Morgan *et al.* 1997; Galvin *et al.* 2003; Robichaud and Stelmach 2003; Evans and Azzopardi 2007). Recently, we used visual masking in a bias-free paradigm to demonstrate that it may not be possible to induce blindsight in normal observers when response bias confounds are controlled for (Peters and Lau 2015); follow-up studies also demonstrated that several other masking techniques commonly assumed to dissociate objective and subjective processing may similarly fail to produce conditions under which blindsight could be induced (Knotts *et al*., 2018).

Yet these failures do not unequivocally prove that blindsight-like behavior *cannot* be induced in normal observers. Although we failed to demonstrate that normal observers can discriminate a stimulus yet be completely unaware of its *overall presence* (Peters and Lau 2015), it may be possible for an observer to be able to discriminate a stimulus above chance while being unaware of its *task-relevant properties*. This might occur especially for stimuli that could possibly bypass “conscious” visual processing areas to activate other task-relevant circuitry, such as observations of amygdala reactivity in “unconscious” face emotion processing (Pessoa and Adolphs 2010; Watanabe and Haruno 2015; Diano *et al.* 2016; Khalid and Ansorge 2017). In such emotion processing “featural blindsight,” the observer would be aware of the face itself and be able to correctly identify its emotion (e.g. happy versus fearful), but would report no subjective or introspective access to the *task-relevant features*, evinced by no introspective confidence in their choices. Indeed, it has recently been reported that processing of emotional stimuli, especially fearful stimuli, may be a powerful candidate for inducing blindsight-like behavior (Vieira *et al.* 2017).

Therefore, here we used emotional face stimuli matched on all low-level properties (e.g. luminance, contrast, spatial frequency) to investigate whether normal observers can identify facial emotions without being aware of their ability to do so. Importantly, we controlled for response bias using the same bias-free confidence paradigm (Mamassian 2020) previously shown to demonstrate optimal introspective access using low-level stimuli (i.e. masked Gabor patches) (Peters and Lau 2015). If we observe emotion-processing “featural blindsight” using this paradigm, the results would suggest that the previous failure to demonstrate blindsight in normal observers was due to the unsuitability of impoverished, low-level stimuli to dissociate objective versus subjective thresholds (Peters and Lau 2015)—and further, that emotion processing may provide an ideal candidate for experimentally isolating subjective awareness from objective task performance capacity in future studies seeking the neural or computational correlates of consciousness.

## Materials and Methods

### Behavioral experiment

#### Participants

Participants for this experiment were recruited from Amazon Mechanical Turk. The task was launched for 40 people; 33 of these participants completed the experiment. Those who completed the experiment were paid $2. Participants were also incentivized to perform well with an extra $1 which was awarded if they performed better than the previous participant (incentive structure is described under Procedure section). Of the 33 people who completed the experiment, 2 had incomplete data, and 2 had >20% of trials which met the exclusion criteria (see below); data from the remaining 29 participants was included in all further analyses. Informed consent was obtained before the start of the experiment, and all procedures were approved by the University of California, Riverside Institutional Review Board and made in accordance with the Declaration of Helsinki.

#### Stimuli

Stimuli consisted of four sets of male and four sets of female faces displaying happiness (“happy” faces), fear (“fearful” faces), or no emotion (“neutral” faces). The faces were selected from the NimStim set of facial expressions (Tottenham *et al.* 2009), which contains images of 43 (18 male, 25 female) people displaying various emotions and neutral expressions. In an earlier experiment (also via Amazon Mechanical Turk), 20 participants viewed 15 female faces and 13 male faces displaying happiness and fear at maximal intensity, and rated the intensity of the emotion shown by each face on a scale of 0–100, where 0 is “completely neutral” and 100 is the “most possible fear/happiness that a person could exhibit.” We selected the four highest-rated faces from each gender for which the difference in the emotional intensity ratings was not significantly different for “happy” versus “fearful” images.

The selected four male and four female faces were then morphed between the “neutral” image and the “happy” and “fearful” images for each person, to create a set of eight morphs from 0% emotional intensity to 100% emotional intensity for each emotion. We then matched the low-level image properties of the morphs using the SHINE toolbox (Willenbockel *et al.* 2010): image luminance histograms of the photos were matched through SHINE toolbox's histMatch function with the structural similarity also optimized through structural similarity (SSIM) gradient ascent (20 iterations), and the amplitude spectrum were matched across images through the specMatch function.

#### Procedure

On each trial, subjects viewed two faces presented sequentially, one after the other. Following the 2-interval forced-choice (2IFC) confidence-betting paradigm (Mamassian 2020) used by Peters and Lau (2015), one interval showed the “neutral” face [Emotion-Absent interval (EA)] and the other showed the same person’s face but with some degree of emotion, selected from the morphed images [Emotion-Present interval (EP)]. The EA and EP intervals were counterbalanced randomly such that the subject would have a 50% of being correct if they bet on either interval. Subjects were asked to determine the emotion of each face (happy/fearful). Subjects were not explicitly informed that one of the intervals had no emotion [which allowed us to compare betting on intervals where there is evidence (EP) versus where there is not (EA)]. Thus, if we find that subjects bet on both EA and EP intervals equally, we can conclude that the metacognitive or introspective confidence—i.e., the awareness of task-relevant stimulus features—for EP intervals is equivalent to that in EA intervals, indicating that subjects had no introspective access to these task-relevant features in the EP intervals. Subjects were also instructed that the two intervals’ emotions were independently chosen, such that any combination of emotion pairs was possible: happy/happy, happy/fearful, fearful/happy, and fearful/fearful. In addition to identifying the emotion, subjects were asked to bet on which emotional identification they felt more confident in. To incentivize their performance, they were told that for each emotion that they identified correctly, they would get a point. They were also told that if they bet on the easier interval, i.e., the one they thought they were more likely to get correct, they would also get a point. These points were then used to determine if they earned the bonus payment.

**Figure 1.**
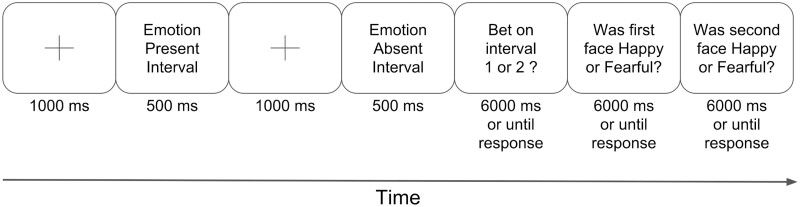
A sample 2-interval forced-choice (2IFC) trial. Subjects viewed two intervals containing a face with Emotion Present (EP interval) or Emotion Absent (EA interval), then “bet” on which emotion discrimination they thought they were more likely to get correct. Subjects then indicated the emotion they identified in the first and second intervals, respectively. The order of the Emotion Present (EP) and Emotion Absent (EA) intervals was counterbalanced between trials.

Each trial began with a fixation point (1000 ms) followed by the first interval’s face presented centrally (size dynamically scaled based on window size to occupy ∼60% of screen height) (500 ms), a second fixation point (1000 ms), and the second interval’s face presented centrally (500 ms) (Fig. 1). The order of EP and EA intervals was counterbalanced across trials, and the emotion to be presented in the EP interval was chosen pseudorandomly such that 50% of trials presented a fearful face and 50% of trials presented a happy face in the EP interval. Each of the two faces used on each trial was chosen randomly from the eight possible face sets, and both faces within a trial were from different sets. For the EP interval in each trial, the emotional intensity of the face (whether happy or fearful) was pseudorandomly set to be 5%, 15%, or 25% for 32 trials each, or 75% for 16 trials (75% is very easy).

After the second interval’s face had disappeared, subjects indicated which interval they felt more confident in (Interval Question), and then the emotion decision (Emotion Questions) for the first and second face, respectively. To indicate their answers, subjects entered their “emotion” choices using the “H” key (happy) and “F” key (fearful) on their keyboard using their right hand, and their choices about which decision to “bet” on using the “1” and “2” keys using their left hand. The next trial began as soon as subjects had pressed a key or after 6 s had elapsed, whichever happened sooner. As described above, only one of the intervals contained an emotional face, with the other containing a neutral face—despite the fact that subjects were told both intervals contained emotional faces.

Before beginning the main experiment, participants completed four easy practice trials and four hard practice trials. Practice trials were identical to trials in the main experiment, except that the faces presented in both intervals were presented for 1000 ms instead of 500 ms, and *both* faces had an emotion present to reinforce the instructions given to subjects to judge the emotion in *both* intervals. Although we could have informed them that only one face contained emotion, we wanted subjects to be equally motivated in both intervals rather than the potential for seeing a clear emotion in the first interval and then not paying attention to the second interval. The emotion intensity in the easy practice trials was 100%, while the emotion intensity in the hard practice trials was 25%. Prior to the beginning of the hard practice trials, participants were told that some emotions would be hard to distinguish, and if they were unsure, they should just make their best guess. After the 8 practice trials, the participants completed 112 trials in the main experiment.

Participants were given two breaks: one after trial 37, and another after trial 74. Each break lasted 1 min, although participants were encouraged to take more time if they needed. In total, the experiment lasted ∼30 min.

#### Analysis

To determine the objective (Type 1) performance capacity for each participant, we calculated the percent of EP trials in which the emotion was correctly identified separately for each emotional intensity level (Emotion Questions). This produced four “% correct emotion discrimination” values for each subject. Following Peters and Lau (2015), we also calculated the confidence/introspective ability of each observer (Type 2) as the percent of trials the subject “bet” on the EP interval at each of the four emotional intensity levels (Interval Question; “% bet on EP”). Trials were excluded from the “% bet on EP” analysis if reaction times to any question (Emotion or Interval Questions) in the trial exceeded 4000 ms, or if the subject failed to respond to all questions in the trial. Likewise, trials were excluded from the “% correct” analysis if reaction time to the Interval Question or the Emotion Question for the EP interval exceeded 4000 ms, or if the subject failed to respond to either of these questions in the trial. We excluded from further analysis any subjects who retained fewer than 80% valid trials after the two exclusion criteria above were implemented.

To look for emotion processing “featural blindsight,” we follow the definitions introduced by Peters and Lau (2015). If subjects show no emotion processing “featural blindsight,” as soon as they are able to meaningfully identify the emotion in the EP interval (“% correct emotion discrimination” > 50%), they should exhibit some degree of introspective access evinced by an ability to bet on their choices (“% bet on EP interval” > 50%); this behavior would match predictions of a Bayesian ideal observer (Peters and Lau 2015). If, in contrast, subjects do exhibit “featural blindsight,” there will be some level of objective performance capacity (“% correct emotion discrimination” > 50% in the EP interval) at which subjects have *no* introspective access to their decisional processes, i.e., *no* ability to meaningfully bet on their choices (“% bet on EP interval” = 50%). To arbitrate between these hypotheses, we compare the goodness of fit of a Bayesian ideal observer computational model to that of a Bayesian observer with additional Type 2 noise, which exhibits featural blindsight. See next sections for details.

During the calculation of *d*′, whenever the subject exhibited a hit rate or false alarm rate of 0 or 1, we applied a standard correction (Wickens, 2001) to ensure that the calculated *d*′ would be within a reasonable range (i.e. not infinity). For the hit rate (HR), we corrected the probability using the formulas

$$\begin{array}{c} If\;HR=0,\;\mathrm{then}\;HR=\frac{1}{\mathrm{nSignal}+1}\\ If\;HR=1,\;\mathrm{then}\;HR=1-\frac{1}{\mathrm{nSignal}+1} \end{array}$$

where *nSignal* is the number of emotion present intervals for that emotional intensity. For the false alarm rate (FAR), we corrected the probability using the formulas

$$\begin{array}{c} If\;\mathrm{FAR}=0,\;\mathrm{then}\;\mathrm{FAR}=\frac{1}{\mathrm{nNoise}+1}\\ If\;\mathrm{FAR}=1,\;\mathrm{then}\;\mathrm{FAR}=1-\frac{1}{\mathrm{nNoise}+1} \end{array}$$

where *nNoise* is the number of emotion present intervals for that emotional intensity.

### Computational model

The Bayesian ideal observer computational model has previously been described elsewhere (Peters and Lau 2015). Briefly, for every 2IFC trial, we draw two samples *d*_EP_ and *d*_EA_, each from a bivariate Gaussian distributions *S*_EP_ and *S*_EA_ (*S ∼ N*(*μ*, *Σ*)) representing an Emotion-Present face and an Emotion-Absent face, respectively. The mean of the generating distribution for the EP face is *μ*_EP_ = [*e*_Happy_, 0] or *μ*_EP_ = [0, *e*_Fearful_], depending on the random assignment of emotional valence in the EP interval; the mean of the generating distribution for the EA face is always *μ*_EA_ = [0, 0]. *Σ* = [1 0; 0 1], the identity matrix, for all generating distributions.

In all cases, the true emotional intensity that generated the emotion, i.e., *e*_emotion_, is unknown to the observer; the observer only has access to the sample that is being observed. This is why the decision about whether each sample is happy or fearful is made according to Bayes’ rule, marginalizing across all possible emotional intensity levels for both Happy and Fearful emotions:

(1) $$p(S,e|d)\;=\frac{p(d|S,e)p(S,e)}{p(d)}.$$

Confidence in the decision (happy or fearful) for each interval is defined as the posterior probability of the choice that was made, i.e.,

(2) $$p(S|e)=\int p\left(S,e|d\right)de.$$

The chosen emotion for each interval, both EP and EA, is defined as the emotion *i* that maximizes this posterior probability, i.e.,

(3) $$S_{\mathrm{chosen}}\;=\;\mathrm{argma}x_{i}\;p\left(S_{i}|d\right)$$

The model then “bets” on the interval that has the higher posterior probability of the choice, i.e., higher confidence, by comparing the posterior probabilities for the chosen emotion in the EP and EA intervals via a decision variable *D*,

(4) $$D=\log\left(\frac{p(S_{\mathrm{chosen},\mathrm{EP}}|d_{\mathrm{EP}})}{p(S_{\mathrm{chosen},\mathrm{EA}}|d_{\mathrm{EA}})}\right).$$

If *D *≥* *0, the model “bets” on the EP interval; if *D* < 0, the model “bets” on the EA interval.

All simulations of this model’s behavior were done via custom-written scripts in Matlab (Natuck, MA), with emotional intensity level *e* ranging from 0.01 to 1 in steps of 0.01 and 5000 samples per emotional intensity level.

#### Simulating emotion processing “featural blindsight”

The ideal observer described above has access to Type 2 information (confidence) with the same degree of fidelity as its access to Type 1 information (emotion decision). In contrast, an observer that exhibits blindsight would have much poorer access to Type 2 information than Type 1 information. Following previous work (Peters and Lau 2015; Maniscalco and Lau 2016), this can be modeled as the addition of increasing amounts of Type 2 noise after the Type 1 decision has been reached. To simulate the behavior of such an observer, we therefore added Gaussian noise to the definition of the Type 2 decision variable *D* after the Type 1 decision, i.e.,

(5) $$D=\log\left(\frac{p(S_{\mathrm{chosen},\mathrm{EP}}|d_{\mathrm{EP}})}{p(S_{\mathrm{chosen},\mathrm{EA}}|d_{\mathrm{EA}})}\right)+\;\epsilon ,$$

where *ε* ∼ *N*(0, *σ*). We simulated predicted behavior for increasing degrees of “featural blindsight” at values of *σ* from 0.01 to 1 in steps of 0.01.

#### Goodness of fit

The goodness of fit of both models—the Bayesian ideal observer and the “featural blindsight” observer at various levels of Type 2 noise (Eq. 5) —was calculated as the multinomial log-likelihood (*L_m_*) of the model with parameters ϕ given the data. This measure quantifies the relative agreement between the data collected from participants and that predicted by a model. We use the following formula:

(6) $$L_{m}(\phi |\mathrm{data})\propto \log\left(\Pi_{ij}P_{\phi }{\left(R_{i}|S_{j}\right)}^{n_{\mathrm{data}}\left(R_{i}|S_{j}\right)}\right),$$

where *S_j_* is the type of stimulus that might be shown on a given trial, and *R_i_* refers to the behavioral response a subject produces on that trial. *n*_data_(*R_i_|S_j_*) represents the count of how many times a human observer produced response *R_i_* after viewing stimulus *S_j_*. Pϕ(*R_i_|S_j_*) represents the model’s prediction of the probability a subject produced response *R_i_* after viewing stimulus *S_j_*, according to the model with parameters ϕ, i.e., the percentage of time the model produced this “response” to this “stimulus.” Importantly, the multinomial log-likelihood quantifies the fit of the *full* distribution of probabilities of each response type given each stimulus type, not just with reference to a summary statistic.

We used this metric to evaluate the predictions of both models at all levels of Type 1 performance exhibited by each human observer. Note that for the ideal observer, there are no free parameters; for the “featural blindsight” observer with Type 2 noise, the only parameter ϕ is the magnitude of the Type 2 noise (Eq. 5).

## Results

Two subjects had incomplete data and our exclusion criteria led us to exclude another two subjects from our analyses, leaving 29 subjects. Across subjects, the stronger three (out of the four) levels of emotion presented typically led to above-chance performance for each emotion intensity: 5% emotion (mean % correct = 49.92 ± SD 0.053), 15% emotion (mean % correct = 0.613 ± SD 0.091), 25% emotion (mean % correct = 0.701 ± SD 0.106), and 75% emotion (mean % correct = 0.897 ± SD 0.139). A one-way repeated measures analysis of variance (ANOVA) showed the expected increase in Type 1 performance (“% correct emotion discrimination”) as a function of increasing emotional intensity [*F*(2.386) = 105.223, *P* < 0.001, Greenhouse-Geisser corrected for sphericity violation; all subsequent cases with Greenhouse-Geisser correction are marked with ^G^]. Likewise, subjects’ ability to bet on the EP interval also increased with increasing emotional intensity as expected (*F*(2.112) = 35.840, *P* < 0.001^G^].

**Figure 2.**
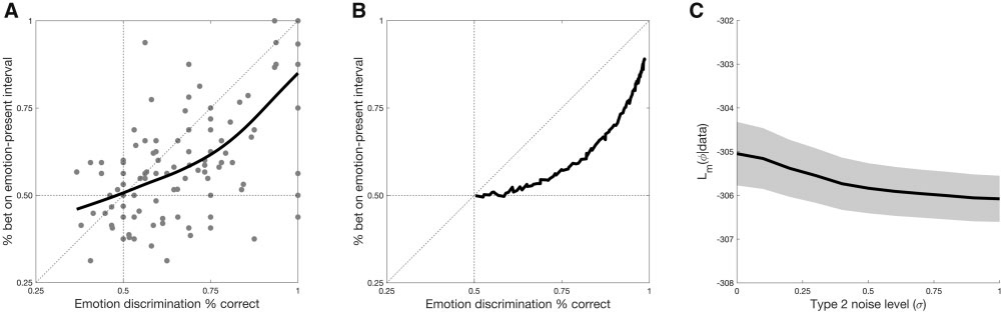
Human subjects show no emotion processing “featural blindsight.” (**a**) As soon as subjects are able to correctly identify the emotion of the face in the Emotion Present (EP) interval (“% correct emotion discrimination” > 50%), they appear to be able to meaningfully bet on their choices (“% bet on EP interval” > 50%). Each point represents one level of emotion for one observer, such that each observer contributes four points to the plot. (**b**) Subjects’ data closely matches predictions from the Bayesian ideal observer computational model. (**c**) Increasing levels of Type 2 noise (*σ*; see Materials and Methods) to produce emotion processing “featural blindsight” in the computational model leads to increasingly *worse* goodness of fit (*L_m_*) between the model and the human subjects’ data [*F*(1.141) = 15.541, *P* < 0.001^G^; two-tailed paired samples t-test between extremes of *σ*  =  0 [no Type 2 noise] and *σ*  =  1 [large Type 2 noise]: *t*(28) = 3.926, *P* ≤ 0.001]. Error cloud represents the standard error of the mean.

The critical analysis is whether subjects show emotion processing “featural blindsight.” Following the definitions introduced by Peters and Lau (2015), if subjects show such emotion processing ability without awareness, we would expect that there would be some level of objective performance capacity (“% correct emotion discrimination” > 50% in the EP interval) at which subjects had no ability to meaningfully bet on their choices (“% bet on EP interval” = 50%). To look for this possibility, we plotted the “% bet on EP interval” values against the “% correct emotion discrimination” values for each subject, and fit a smoothing spline [using Matlab’s smoothingspline function, which minimizes the expression $p\sum_{i}w_{i}(y_{i}-s(x_{i}){)}^{2}+(1-p)\int {\left(\frac{d^{2}s}{dx^{2}}\right)}^{2}dx$, with the smoothing parameter set to 0.99] to the data across all subjects (Fig. 2a). Data from individual subjects closely resembles the group data (Supplementary Fig. S1), and analogous analyses using *d*′ in the EP interval (*d*′EP) and *d*′ in selecting the interval containing the emotional face (*d*′2IFC) revealed similar results (Supplementary Fig. S3).

From visual inspection alone, we see no evidence of emotion processing “featural blindsight”: as soon as subjects are able to correctly identify the emotion in the EP interval, they are able to meaningfully bet on their choices to some degree. The pattern of the data closely mimics the failure to observe blindsight-like behavior in normal observers using low-level visual stimuli, in which the target-absent interval (here the EA interval) contained no stimulus at all (Peters and Lau 2015). It also visually matches predicted behavior from a Bayesian ideal observer (Fig. 2b).

However, because this study was done online through Amazon mTurk, the data for individual subjects is somewhat noisier than it would be had subjects each completed many hours of psychophysics, as they did in the original Peters and Lau (2015) study (Supplementary Fig. 1). Therefore, we rely on the quantitative goodness of fit metrics to critically evaluate whether any hint of “featural blindsight” might be present.

We calculated the goodness of fit as *L_m_* for each human observer for the Bayesian ideal observer, and compared it to *L_m_* for the “featural blindsight” observer model at each level of Type 2 noise (see Materials and Methods, Eq. 6). This analysis revealed that as Type 2 noise increases—i.e., as “featural blindsight” becomes stronger—the model fits the data less and less well [main effect of Type 2 noise magnitude, *F*(1.141) = 15.541, *P* < 0.001^G^; Fig. 2c]. This interpretation was confirmed with a two-tailed paired samples t-test of the log likelihoods between *σ  = * 0 (no Type 2 noise, i.e., Bayesian ideal observer) and *σ*  =  1 (high Type 2 noise) confirms that goodness of fit decreases as Type 2 noise increases [*t*(28) = 3.926, *P* ≤ 0.001]. Thus, the Bayesian ideal observer with no Type 2 noise—i.e. the observer with no “featural blindsight”—provides the best explanation of the human subjects’ behavior.

## Discussion

Here, we used a bias-free two-interval forced-choice (2IFC) method to examine whether facial emotion processing might reveal blindsight-like behavior in normal human observers. We modified a previous version of this paradigm (Peters and Lau 2015) to match low-level visual properties such as luminance, contrast, and spatial frequency differences between the Emotion Present (EP) and Emotion Absent (EA) intervals; our goal was to reveal emotion processing “featural blindsight,” in which observers would be able to correctly identify an emotion when it was present but have no ability to introspect on the evidence or process leading to their choices. However, despite previous reports suggesting that emotion processing may bypass conscious visual processing areas (Pessoa and Adolphs 2010; Watanabe and Haruno 2015; Diano *et al.* 2016; Khalid and Ansorge 2017) and that emotional (especially fearful) stimuli may be powerful candidates for inducing blindsight-like behavior (Vieira *et al.* 2017), here we saw no evidence to suggest that emotion-processing “featural blindsight” may occur.

These results are in line with the absence of evidence for blindsight-like behavior reported in the original Peters and Lau (2015) study (which used forward–backward masking and simple visual stimuli), as well as the observation that various masking techniques fail to produce differences in the relationship between objective versus subjective thresholds (Knotts *et al*., 2018). Our observations also support the finding that even noninvasive transcranial magnetic stimulation fails to produce the ability to discriminate a stimulus in the absence of any visual awareness or confidence (Peters *et al.* 2017). Taken together, the evidence appears to be mounting that it is extremely difficult—if not impossible—to use visual masking or noninvasive techniques to produce dissociations between performance and awareness that would be ideal for experimentally isolating subjective consciousness.

However, despite the consistency of the present results with previous reports, it should certainly be noted that the data collection method we used here was quite different from the standard approach. Each of our subjects completed only 112 trials in the main experiment across four levels of emotion intensity, which is significantly less than the volume of data collected in e.g. the original study (2600 trials; Peters and Lau 2015). One could argue that the online data collection method, with so few trials per subject, may preclude the possibility of detecting the blindsight-like effect we hoped to reveal. In response, we note that each of our 29 subjects’ data looks much like the data in aggregate, and that the goodness of fit metrics were calculated for each subject individually rather than for the group average. That we identified decreasing goodness of fit for increasing Type 2 noise, despite the noisiness of online data collection and the small trial numbers, suggests that the data collection method was not so noisy as to prevent the possibility of identifying emotion processing “featural blindsight.” Future studies may wish to follow-up the results presented here with additional variations, done both in the laboratory and in an online setting.

Another way to check whether our online data collection method produces meaningful data would be to compare the emotional discrimination results to previous reports. In another study of emotion processing and confidence, it was reported that fear processing is “special”: when perceiving a fearful face human subjects show a liberal bias in both detection and discrimination tasks, and “fear” choices are also reported with higher confidence (Koizumi *et al.* 2016). We used signal detection theoretic approaches (Green and Swets 1966; Macmillan and Creelman 2004) to examine whether the same biases would be present in our own data. Consistent with previous reports that fearful faces are perceived more easily or quickly (Milders *et al.* 2006; Phelps *et al.* 2006; Stein *et al.* 2009, 2010, 2014; Stienen and de Gelder 2011; Amting *et al.* 2010), we also saw a significant shift in the Type 1 decisional criterion that biased subjects to respond “fearful” more than “happy” at lower emotional intensities in both the EP [*t*_5%_(28) = 4.174, *P* < 0.001; *t*_15%_(28) = 3.917, *P* < 0.001; *t*_15%_(28) = 2.040, *P* = 0.051; 75% intensity N.S.; 5% and 15% intensities are significant with Bonferroni correction for multiple comparisons] and EA [*t*(28) = 5.219, *P* < 0.001] intervals. Interestingly, the magnitude of this shift when significant or trending (*c*_EA_ = −0.600, *c*_5%_ = −0.444, *c*_15%_ = −0.352, *c*_15%_ = −0.187) was somewhat larger than that reported by Koizumi *et al.* (2016) in their discrimination task (*c* ≈ −0.12). This result provides further evidence that online data collection, despite being impoverished relative to the controlled setting of the laboratory, may be a viable method for identifying even small effects.

It is interesting to note that the Bayesian ideal observer here, as in Peters and Lau (2015), may appear to show some level of performance without awareness—here in the form of featural blindsight as well—at low levels of performance, just above chance (Fig. 2b). However, we reiterate that this apparent performance without awareness is really due to a lack of statistical significance in the ability to “bet” on its choices as being different from chance in this very low region of the performance curve. In the present manuscript we implemented the Bayesian ideal observer with Monte Carlo simulations with a finite number of simulated trials for convenience, but mathematically, $p(S_{\mathrm{chosen}}|d)>0.5$ means that a Bayesian Ideal Observer with infinite compute – which could represent the entire posterior probability distribution—can “bet” above chance exactly as soon as it can discriminate a stimulus’ identity above chance. The appearance of performance without awareness for the ideal observer therefore only comes from limitations of the simulation.

Given the nature of the 2IFC task used here in the subjective rather than objective task (de Gardelle and Mamassian 2014; Peters and Lau 2015; de Gardelle *et al.* 2016; Mamassian 2020), it is important to clearly discuss how interval betting and emotion discrimination relate to constructs of subjective versus objective processing and the impact of these relationships on the interpretation of results. Here, interval betting is used to index subjective awareness because it relates to metacognition and 2IFC detection (Mamassian 2020) [although it should not be taken to mean that we equate awareness and subjective confidence (Rosenthal 2019); in contrast, emotion discrimination capacity provides an index of perceptual performance. We acknowledge that the relationships between these measures and the constructs they assume to quantify in the present project are not necessarily universally agreed-upon, especially when it comes to the relationship between metacognition and awareness (Nelson 1996; Rosenthal 2019). Yet these concepts have become increasingly tightly coupled both empirically and theoretically (Brown *et al.* 2019; Lau 2019; Lau and Brown, 2019), so despite their theoretical distinction it can be argued that metacognitive judgments such as those used here provide a sufficient indicator for conscious awareness in experimental contexts.

The present results—and previous studies showing similar failure to induce blindsight-like behavior using generally accepted methods for doing so (Peters and Lau 2015; Peters *et al*. 2017a)—present a growing challenge to the dominant view that stimulus manipulation and/or noninvasive brain stimulation may result in task performance capacity in the absence of introspective access (Merikle 1982; Reingold and Merikle 1988; Kolb and Braun 1995; Merikle *et al.* 2001; Boyer *et al.* 2005; Charles *et al.* 2013). We acknowledge that the growing body of work we add to here does not speak definitively to the *impossibility* of inducing dissociations between the objective and subjective thresholds in normal human observers—i.e., they do not support the possibility of inducing *d*′ > 0 (above objective threshold) without accompanying awareness (below subjective threshold). However, these results do increasingly suggest that inducing blindsight-like perceptual capacity in the absence of introspective access may be much more difficult to induce than commonly believed (Heeks and Azzopardi 2015). Consequently, demonstrating such a dissociation using this conservative 2IFC confidence method would provide indisputable evidence that blindsight *can* be induced in normal observers if one employs the appropriate experimental manipulations.

## Supplementary data

Supplementary data is available at NCONSC Journal online.

## Data accessibility statement

The code used in the simulations and data presented here can be found at https://github.com/vrsivananda/Faces_2IFC_Task.

*Conflict of interest statement*. None declared.

## Supplementary Material

niaa023_Supplementary_Data
