# Supplementary material for: Normal observers show no evidence for blindsight in facial emotion perception
Source: Neurosci Conscious. 2020 Dec 12;2020(1):niaa023. doi: 10.1093/nc/niaa023 (PMC7734439; doi:10.1093/nc/niaa023)
Supplement: niaa023_Supplementary_Data [file niaa023_supplementary_data.docx]

**Supplemental Material**

Normal human observers show no blindsight in facial emotion perception

Sivananda Rajananda, Jeanette Zhu, Hakwan Lau, & Megan A. K. Peters

**Individual subjects’ data**


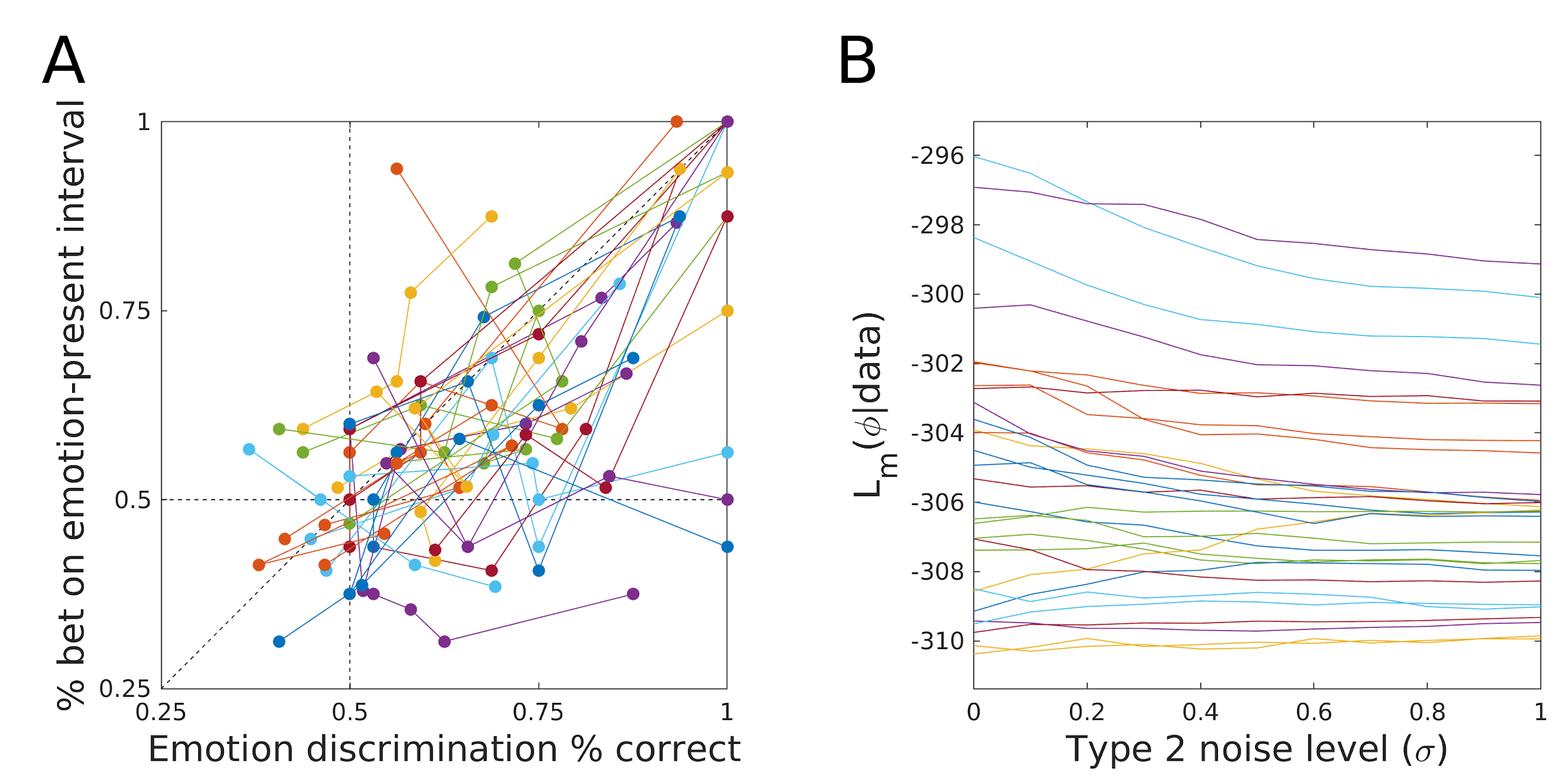


Figure S1. Individual subjects’ behavioral results and goodness of fit. (a) Individual subjects’ results closely mimic aggregate results, demonstrating no convincing evidence for emotion processing ‘featural blindsight’. As soon as subjects are able to discriminate the Emotion Present interval’s emotion above chance, they have some ability to bet on their choices, as predicted by a Bayesian ideal observer. (b) As Type 2 noise increases for the ‘featural blindsight’ observer model, the goodness of fit of the model to the data decreases. This shows that of the models we tested, the Bayesian ideal observer with no ‘featural blindsight’ provides the best fit to each individual subject’s behavior. In both panels, each color represents one individual subject’s data.


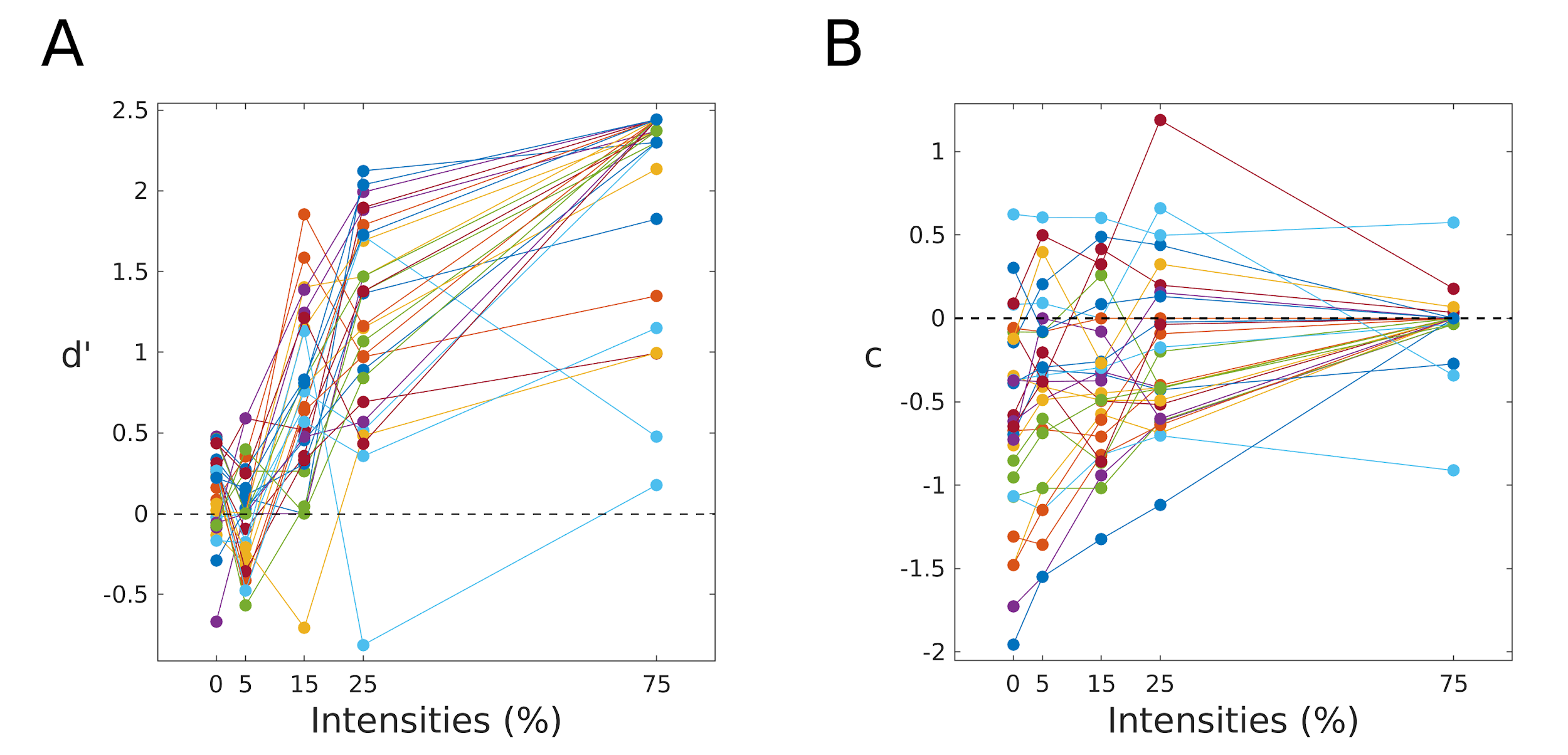


Figure S2. Type 1 performance (d’) and response bias (*c*) as a function of stimulus emotion intensity. (a) As emotion intensity increases, subjects show the expected increase in objective performance capacity. (b) Subjects exhibit significant bias to respond ‘fearful’, especially at low emotion intensities. As above, in both panels, each color represents one individual subject’s data.

Alternative performance metrics

Given the known relationship between 2-interval forced-choice (2IFC) discrimination d’ and yes-no detection d’ (by a factor of $\sqrt{2}$) [(Azzopardi & Cowey, 1997; Evans & Azzopardi, 2007; Heeks & Azzopardi, 2015)](https://paperpile.com/c/FdcRlh/3xEj+uOSw+J8fH), we also examined whether blindsight might be revealed by using d’ as a metric of sensitivity instead of percent correct as in the main text. Although the EP interval yes-no discrimination was between happy-angry and the 2IFC discrimination was between emotion-present versus emotion-absent, we still endeavored to examine whether d’_EP_ would approach d’_2IFC_/$\sqrt{2}$ or whether the d’ metrics might reveal blindsight-like behavior (Figure S3). However, just as in the main analysis, we observed no hint of blindsight-like behavior: as soon as d’_EP_ rose above chance (d’_EP_ = 0), d’_2IFC_ also departed from chance and approached the ideal observer optimal $\sqrt{2}$ factor of d’_EP_.


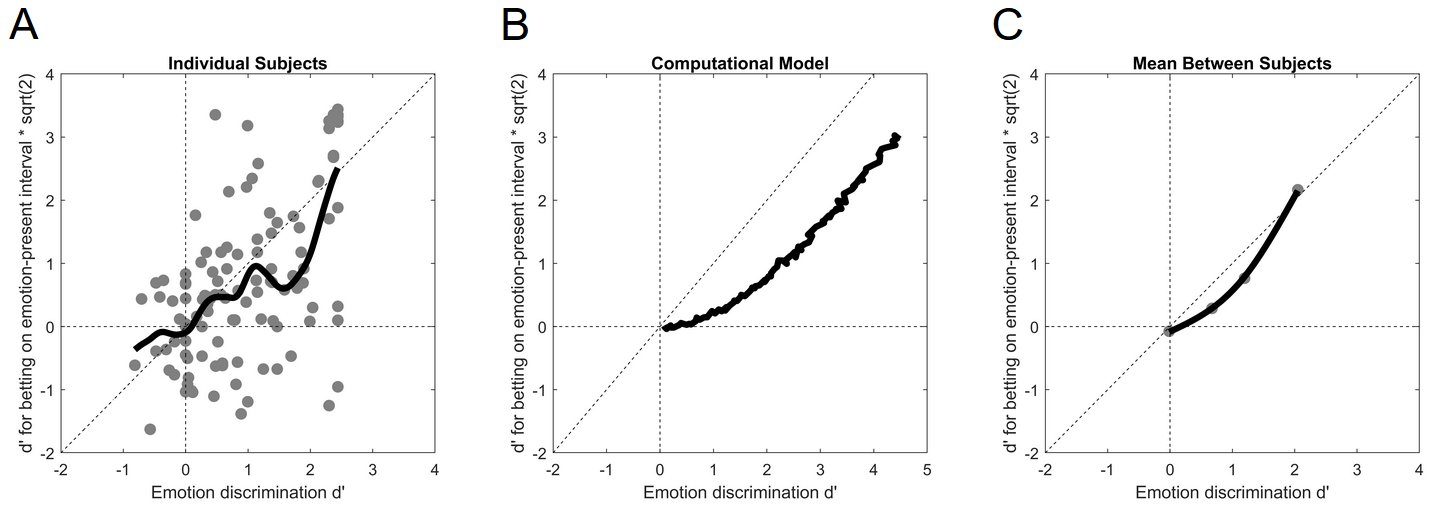


Figure S3. Emotion discrimination d’ versus 2IFC betting d’, corrected by factor of $\sqrt{2}$.
